# Prevalence of nosocomial infections and anti-infective therapy in Benin: results of the first nationwide survey in 2012

**DOI:** 10.1186/2047-2994-3-17

**Published:** 2014-05-14

**Authors:** Théodora Angèle Ahoyo, Honoré Sourou Bankolé, Franck Mansour Adéoti, Aimé Attolou Gbohoun, Sibylle Assavèdo, Marcellin Amoussou-Guénou, Dorothée Akoko Kindé-Gazard, Didier Pittet

**Affiliations:** 1 Génie de Biologie Humaine, Ecole Polytechnique d’Abomey-Calavi, University of Abomey-Calavi, Cotonou, Benin; 2 International Network for Planning and Improving Quality and Safety in Health Systems in Africa (Réseau International pour la Planification et l’Amélioration de la Qualité et la Sécurité dans les établissements humains en Afrique), Abidjan, Ivory Coast; 3 Improvement Quality Service, Ministry of Health, Cotonou, Benin; 4 Ministry of Health and Faculty of Medicine, University of Abomey-Calavi, Cotonou, Benin; 5 Infection Control Programme and WHO Collaborating Centre on Patient Safety, University of Geneva Hospitals and Faculty of Medicine, Geneva, Switzerland

**Keywords:** Nosocomial infection, Prevalence, Antimicrobial resistance, Co-morbidity, Antibiotic use, Infection control, National surveillance, Surveillance, Africa, Low-/middle-income countries

## Abstract

**Background:**

Data on nosocomial infections in hospitals in low-income countries are scarce and often inconsistent. The objectives of this study were to estimate the prevalence of nosocomial infections and antimicrobial drug use in Benin hospitals.

**Methods:**

All hospitals were invited to participate in the first national point prevalence study conducted between 10–26 October 2012 using the protocol developed by the “Hospitals in Europe Link for Infection Control through Surveillance” (HELICS) project. Infection prevalence rates and the proportion of infected patients and exposure to antimicrobials were assessed.

**Results:**

Overall, 87% (39/45) of hospitals participated. Of 3130 inpatients surveyed, 972 nosocomial infections were identified among 597 patients, representing an overall prevalence of infected patients of 19.1%. The most frequent infections were related to the urinary tract (48.2%), vascular catheter use (34.7%), and surgical site (24.7%). 64.6% of patients surveyed were treated with antibiotics, including a significant proportion (30%) of non-infected patients and a high proportion of self-medication (40.8%). Resistance of leading nosocomial pathogens to antimicrobials included methicillin-resistance (52.5%) among *Staphylococcus aureus*, vancomycin resistance among enterococci (67.5%), cefotaxime resistance among *Escherichia coli* (67.6%), and ceftazidime resistance among *Acinetobacter baumannii* (100%) and *Pseudomonas aeruginosa* (68.2%).

**Conclusions:**

Benin has high nosocomial infection rates and calls for the implementation of new national infection control policies. Patient safety education and training of all individuals involved in healthcare delivery will be critical to highlight awareness of the burden of disease. The high use of antimicrobials needs to be addressed, particularly their indiscriminate use in non-infected patients.

## Introduction

Nosocomial infections, also named healthcare-associated infections (HAI), are a global problem in every hospital around the world. Quantification of HAI is needed to help justify resources dedicated to infection control. Recognition of the problem and its characteristics vary considerably from country to country [1-3], but the “Hospitals in Europe Link for Infection Control through Surveillance” (HELICS) project has resolved this issue by creating a consensus prevalence protocol [4].

Hospital infection control practices remain rudimentary in the developing world, mostly due to severely limited resources [5,6]. Furthermore, the concept of interrupting HAI transmission takes on another meaning when patients’ relatives have to take up temporary residence in the hospital to provide food, care, and comfort [5,7]. Reports of HAI rates in African countries are scarce, particularly at national level [6,7]. However, several reports in the literature of the occurrence of HAI outbreaks and hospital-wide prevalence surveys have revealed rates ranging between 2.5% and 14.8% [8,9]. Data on causative pathogens are available from a few studies only and highlight the importance of Gram-negative rods, particularly in surgical site infections [10,11], and coagulase-negative staphylococci (CNS) responsible for bloodstream infection [11].

HAI surveillance helps to assess the magnitude of disease in populations and constitutes the first step to prevention [12,13]. We describe the results of the first national prevalence survey of HAI and antimicrobial resistance in Benin and provide a global picture of the epidemiological situation in this country.

## Material and methods

### Study design

A national research group designed a one-day point prevalence survey in collaboration with international experts. The invitation to participate was sent in July 2012 to all hospital directors in Benin. Participation was voluntary, but strongly encouraged. To ensure consistency across all hospitals, it was decided to define HAI by the presence of clinical symptoms and signs not laboratory results only. The United States Centers of Disease Control and Prevention (CDC) criteria [13] were used to identify HAIs, based on the presence of symptoms. Asymptomatic bacteriuria was not recorded as a HAI.

The survey was conducted from 10 to 26 October 2012; patients from the same ward were surveyed the same day in each hospital. Data were collected by nurses and epidemiologists trained for this purpose [13,14] by applying the HELICS project prevalence protocol [4]. The feasibility of the approach was assessed in a pilot study conducted in March 2012. Patient characteristics and types of HAI were recorded on case report forms. Only HAIs occurring in the acute hospital setting were considered, thus excluding for example, long-stay psychiatric wards and day care activities, such as the outpatient day clinic. Infections developing after discharge were not included.

The study protocol was approved by each local ethics committee. Due to the observational character of the study, written informed consent was not required.

### Microbiology

Bacterial samples were collected as recommended by Freney et al. [15]. *S. aureus* strains were primarily investigated for morphological and biochemical characteristics, including Gram stain, aerobic, anaerobic optional, catalase, acetone, and coagulase-producing. Standard microbiological methods for identification of microorganisms were applied for others.

Antimicrobial susceptibility testing was performed by agar diffusion method on Mueller Hinton agar (Bio-Rad-Diagnostic Pasteur, Marnes la Coquette, France) as recommended by the United States Clinical and Laboratory Standards Institute (CLSI) [16]. Interpretation of antimicrobial susceptibility followed the recommendations of the Antibiogram Committee of the French Society for Microbiology Society. Evaluation of methicillin resistance was performed by plating the strains on buffered Mueller-Hinton (Bio-Rad-Diagnostic Pasteur) with NaCl 2% (wt/vol) at 37°C for 24 h.

### Statistical analysis

A distinction was made between the prevalence of patients infected (several infections by the same patient were counted only once) and the prevalence of HAI (several infections by the same patient were counted separately). All statistically significant variables were analyzed using a logistic regression model to demonstrate associations between individual variables and organism acquisition. Two-tailed p values of < 0.05 were considered statistically significant. All statistical analyses were performed using SPSS software (SPSS 19.0 for Windows).

## Results

### Overall results

Data were collected from 39 of 45 healthcare facilities (87%) and represented a total of 3761 beds, corresponding to 65% of the full capacity of participating centres. Of the 3130 inpatients surveyed, there were 972 infections among 597 patients. The average prevalence of infected patients was 19.1% (95% confidence interval [CI], 15.8-22.4) and the overall prevalence of infection was 31.0% (95% CI, 21.8–35.2). Table 1 shows the average prevalence of infected patients according to the type of hospital and geographical region.

**Table 1:** Prevalence of patients infected by hospital type and region; 1st National Prevalence study, Benin, October 2012

**Subgroup**	**Patients surveyed (N)**	**Patients infected (N)**	**Prevalence of infected patients (%)**
ALL	3130	597	19.1
**By region**			
North region	1175	353	30.0
South region	917	165	18.0
Central region	1038	79	7.6
**By hospital type**			
Departmental	642	134	20.9
General hospital	1801	323	17.9
University hospital	665	132	20.5

Surgical (182 cases; 30.5% of all HAI), internal medicine (135 cases; 22.6%), and obstetrics and gynaecology wards (115 cases; 19.3%) were the three services most affected by HAI. On the days surveyed, 11% of patients had been hospitalized for three days or less, 45% between 4 to 14 days, and 36% for more than 15 days. Information was not available for 8%. Overall mortality among infected patients was 22% (131/597) within seven days following infection onset versus 7.4% (189/2533) among non-infected patients (odds ratio [OR], 2.9; 95% CI, 2.1-3.2).

### Infection sites and microbiological results

The most frequent infections were urinary tract infection (37.5%), intravascular catheter-associated (27%), and surgical site infection (19.2%), followed by lower respiratory tract infection or pneumonia (11.7%), bloodstream infection (1.5%), and infections of other origins (3.1%). A total of 972 microorganisms were identified during the study period; 65% (n = 632) were Gram-negative, 30% (n = 291) were Gram-positive, and 5% (n = 49) others. Most isolates were obtained from pus (44.3%), urine (43.5%), and blood cultures (10.1%). Table 2 shows the most frequently encountered microorganisms.

**Table 2:** Distribution of the most frequently identified nosocomial pathogens; 1st National Prevalence study, Benin, October 2012

**Microorganism (no. of isolates)**	**(%)**
*Staphylococcus aureus* (271)	27.9
*Escherichia coli* (221)	22.7
*Pseudomonas aeruginosa* (110)	11.3
Enterococci (102)	10.5
Other GNR (99)	10.2
CNS (47)	4.9
*Salmonella* spp. (41)	4.2
Undetermined (30)	3.1
*Citrobacter* spp (26)	2.7
*Candida* spp (15)	1.5
*Acinetobacter baumanii* (10)	1
**Total (972)**	**100**

GNR, Gram-negative rod-shaped bacteria not further identified;

CNS, coagulase-negative staphylococci.

### Patient characteristics

Characteristics of infected patients (n = 597) show a bimodal age distribution with peaks at the extremes of age. In the 15 to 44-year-old age group (53.8%), mean age was 26 years and infected patients were mostly female. By contrast, no such difference was observed among patients older than 55 years (mean age, 62 years). Within these 597 patients, 59.3% were female, 40.7% were male, 34% have married status, 32% single, 10% divorced, 7% widowed, and 17% of unknown marital status. The occupation of infected patients showed that most were engaged in agricultural activities (40.4%), followed by civil servants (13.6%), household services (12%), and traders (4.5%); 29.5% were without any occupation.

At the time of HAI identification, 55% of 597 patients had been admitted from home, 29% from another service in the same hospital, and 16% from another hospital. Multivariate logistic regression analysis showed that the risk of developing a HAI was mainly related to catheter use (OR, 1.7; 95% CI, 1.2-2.9), duration of stay (OR, 8.2; 95% CI, 5.4-9.3), and inappropriate (unneeded and non-prescribe) use of antibiotics (OR, 5.7; 95% CI, 3.8- 7.4).

### Antimicrobial susceptibility

Tables 3 and 4 show the antimicrobial susceptibility patterns of the most frequently isolated bacterial pathogens. The frequency of resistance to methicillin among *S. aureus* isolates was high (52.5%); the frequency of resistance to vancomycin among *S. aureus* isolates was 7.7%, and the frequency of resistance to vancomycin among enterococci was 67.5%.

**Table 3:** Resistance patterns of leading Gram-positive bacterial isolates to main antimicrobial agents; 1st National Prevalence study, Benin, October 2012

**Antimicrobial**	***S. aureus *** (271*)	CNS (47*)	Enterococci (102*)
Penicillin	98%	95%	68%
Oxacillin	52.5%	35%	_
Ampicillin	_	_	62.7%
Amoxicillin clavulanate	55%	23%	23%
Tetracycline	85%	78%	74%
Erythromycin	37%	54%	26%
Chloramphenicol	36%	32%	18%
Gentamicin	45%	35%	62.3%
Trimethoprim sulfamethoxazole	64%	70%	51%
Vancomycin	4%	1%	67.5%

*Number of isolates tested.

**Table 4:** Resistance patterns of leading Gram-negative bacterial isolates to main antimicrobial agents; 1st National Prevalence study, Benin, October 2012

**Antimicrobial**	***E. coli *** (221*)	***Salmonella *** (41*)	***P. aeruginosa *** (110*)	***Citrobacter *** (26*)	***A. baumanii *** (10*)	Other Gram-negative (99*)
Ampicillin	74	78	100	89	100	76%
Amoxicillin clavulanate	72.8%	70%	90%	76%	100%	56%
Ceftazidime	60%	55%	67.5%	64%	100%	63%
Tetracycline	78%	75%	77.3%	88%	80%	95%
Gentamicin	45%	35%	22%	26%	75%	9.5%
Trimethoprim sulfamethoxazole	65%	68%	64%	56%	62%	87%
Ciprofloxacin	18%	13%	22%	15%	16%	9%
Imipenem	0	0	0	0	0	0

*Number of isolates tested.

The highest proportions of resistance among Gram-negative isolates were observed against ampicillin (86.4%), tetracycline (77.3%), amoxicillin/clavulanic acid (72.8%), and trimethoprim/sulfamethoxazole (64%). There were no clear-cut differences in antimicrobial susceptibilities among the various serotypes of *Salmonella* isolates. Most *Pseudomonas aeruginosa* isolates produced an extended spectrum beta-lactamase and were resistant to all potentially active beta-lactams, with the exception of imipenem (second-line drug not currently available in Benin); only 24 isolates (22%) were susceptible to gentamicin and ciprofloxacin. The main resistance patterns observed among identified pathogens are summarized in Table 5.

**Table 5:** Major resistance patterns identified among bacterial isolates; 1st National Prevalence study, Benin, October 2012

	
MRSA (n = 142/271)	52.5%
MRSA vancomycin R (n = 11/142)	7.7%
Enterococcus ampicillin R (n = 64/102)	62.7%
Enterococcus vancomycin R (n = 69/102)	67.6%
*E. coli* cefotaxime R (n = 89/221)	40.2%
*E. coli* ESBL + (n = 44/221)	20.0%
*Acinetobacter baumanii* ceftazidimeR (n = 10/10)	100.0%
*Pseudomonas aeruginosa* ceftazidime R (n = 75/110)	68.2%

R, resistant; MRSA, methicillin-resistant *S. aureus*;

ESBL, extended-spectrum beta-lactamase.

### Anti-infective treatment

The prevalence of anti-infective therapy was 64.6% (2023/3130); 86.2% (514) of infected patients and 30% (760) of non-infected patients received antimicrobials. The remaining (13.8%) of infected patients did not receive any antibiotic treatment. More than 35% of treated patients were receiving more than one antimicrobial. A total of 4130 drugs were prescribed during the study period. Of these, 6% were prescribed under their international nonproprietary name. One-third of prescriptions (37.4%; 1547/4130) of an antibiotic resulted from a doctor’s prescription. In 40.8% (1686/4130) of cases, the drug was used as self-medication (without doctor’s prescription). Details of antimicrobials used are shown in Table 6.

**Table 6:** Most frequently used antimicrobials; 1st National Prevalence Study, Benin, October 2012

**ANTIBIOTICS**	**%**
**Beta-lactam**	**86.9**
Ampicillin, cloxacillin, amoxicillin, amoxicillin-acid clavulanic	
**Cephalosporin**	**17.4**
Ceftriazone, cefotaxime, ceftazidime	
**Fluoroquinolone**	**8.5**
Ciprofloxacin, ofloxacin, peflacin	
**Imidazole**	**7.5**
Metronidazole, tinidazole	
**Aminoside and sulfamides**	**6**
Gentamycin, netromycin, trimetroprim-sulfamethazole	
**Macrolides**	**1,5**
Erythromycin, spiramycin, azithromycin	

## Discussion

The first national point prevalence study was organized in the context of the 1st International Conference of Health Ministers of Africa (CIMSEF 2012) held in Cotonou, Benin, from 10–12 December 2012 as data from Benin were not available. The one-day prevalence survey methodology was selected as the most effective way to generate necessary data in a short time and with the potential to provide valuable information to guide future infection control interventions using limited resources. Participation was high and the global estimated coverage was approximately 87% of all hospitals in Benin, thus constituting a representative sample of acute care institutions in terms of region, type of wards, hospital size, and teaching status. The total number of patients surveyed was 3130, corresponding to 65% of the full capacity of participating centres. Occupancy rate did not reach 100%.

Although the overall bed occupancy rate did not reach 100%, some hospitals exceeded their bed capacity occasionally with more than one patient occupying the same bed [3,5].

We observed an overall HAI prevalence of 19.1%. Surgical and medical wards showed high rates (22.3% and 24.5%, respectively) and comprised approximately half of the total number of HAIs observed. These findings are higher than previously reported by Bagheri Nejad et al. and Simon et al. [5,8]. The difference might be accounted for by different diagnostic strategies and criteria used. The availability and use of microbiology laboratory data contribute also to the increased identification of HAI. These rates are much higher than in developed countries, where they range from 5-10% [11,12] and well illustrate the urgent need to identify and implement feasible and sustainable approaches to strengthen HAI prevention, surveillance, and control in Africa.

The successful application of the HELICS project protocol to determine the presence of HAI patient symptoms in a standardized manner represents a remarkable aspect of our study. This method, often employed in Europe, was easy to use and is probably more accurate and less variable than previous methods, which relied heavily on interpretation by the local observer and could have contributed to higher estimates. Despite the high prevalence rate, most infected patients probably go undiagnosed, mainly due to the lack of routine surveillance and absence of facilities for microbiological diagnosis. This reference protocol has some limitations for use in developing countries. First, the available resources are modest and the cultural context is different. Second, the classical community-acquired infectious diseases, such as typhoid fever, are largely controlled in developed countries [16,17], but continue to be a common occurrence in most African countries.

Staphylococci were the most prevalent bacteria and were isolated from almost all types of clinical samples considered. This result is not surprising because of its opportunistic and ubiquitous nature [18]. *S. aureus* was the most common hospital-acquired pathogen among inpatients in our study, followed by *Escherichia coli*, *P. aeruginosa*, and enterococci.

*Clostridium difficile* is well established as important bacteria in HAI in Benin. Its presence is usually associated with a variety of clinical manifestations and also we were limited by the availability of diagnostic tests that indicate the presence of this microorganism.

The prevalence of antibiotic use was 64.6%, similar to published data from Tanzania (71%) or Greece (51.4%), but much higher than the rate reported from France (16%) [12,19]. The most extensive use of antimicrobials in non-infected patients was in surgical (27%) and obstetrics/gynaecology (18%) wards.

In our study, the treatment of 13,8% of infected patients was a challenge by the fact that the prohibitive cost of second-line antimicrobial drug, when available, places them out of the reach of majority of patients.

It is imperative to preserve the effectiveness of common antibiotics by promoting their rational use based on sound knowledge of local resistance patterns.

Several resistant strains were identified in agreement with previously reported findings [19,20], e.g., methicillin-resistant *S. aureus* (52.5%), ceftazidime-resistant *P. aeruginosa* (68.5%), and vancomycin-resistant enterococci (67.5%). This might be due to the fact that certain classes of antibiotics are easily accessible in Benin and frequently used by patients without a medical prescription; this represents 40% of patients on antibiotics. In our context the self-medication was related both to other illnesses and HAI treatment.

These results are in acccordance with data reporting the increasing antimicrobial resistance of Gram-negative bacteria [19,21,22]. In addition, the high proportion of multiresistant bacteria in Benin may be explained by the practice of patient self-medication and the availability and low price of antibiotics that can be bought without prescription. An additional concern is the fact that some available pharmaceuticals are of doubtful quality and fail basic quality tests, thus raising fears that many of these drugs may not be having the desired impact on patients. It is therefore critical to treat HAI appropriately by starting antimicrobial therapy early in the course of infection, using the correct agent at the most appropriate dose, and for an adequate duration. Furthermore, antibiotic stewardship should include patient education.

Of note, no resistance to imipenem was recorded among pathogens identified in this nationwide survey of HAI. In Benin, the repertoire of first-line drugs is limited to ampicillin, chloramphenicol, erythromycin, gentamicin, penicillin, tetracycline and trimethoprim-sulphathoxazole. Second-line antibiotics include imipenen, which is currently unavailable in Benin. Furthermore, when available, its prohibitive cost would place it out of the reach of most patients. Thus, the lack of use explains the absence of identified resistance to imipenem in Benin.

Our study has limitations. First, and similar to all point-prevalence studies, this approach tends to underestimate true prevalence rates and, in particular, HAI of shorter duration. In addition, HAI that developed after hospital discharge was not recorded. A realistic picture of the disease pattern over time would require the conduct of prospective surveillance of HAIs, which is far beyond available resources in Benin. Second, antibiotic resistance detection was not conducted at a central laboratory, but the surveillance method employed did use international standardized techniques. As multiresistant nosocomial pathogens of high epidemiological interest (i.e., vancomycin-resistant *S. aureus*) were not kept, further diagnostic validation could not be conducted. Differences in the sensitivity and specificity of laboratory diagnostic techniques have been described, which might impact on stain identification [23].

To our knowledge, this is the first study that provides national prevalence data for HAI in Benin. Moreover, the methodology proved feasible in the African setting and is probably more accurate than previous methods used. In the light of these findings, appropriate prevention measures can be proposed, such as reminders regarding hygiene rules and recommendations and isolation protocol, completed by HAI surveillance. We suggest that the combination of a prevalence (cheap and short, and providing a one-day snapshot) and an incidence survey (targeted surveillance of high-risk areas) provide the best picture of HAI in settings with limited resources.

## Conclusions

Our results suggest that the prevalence of HAI in Benin is at least as high as published reports from neighbouring countries and that continued infection control efforts are warranted. This is also the first report of national prevalence of HAI in Benin. We believe there is a need for a standardized approach to HAI surveillance in Africa region. More attention should be paid to the indiscriminate use of antimicrobials in non-infected patients.

## Competing interests

The authors declare that they have no competing interests.

## Authors’ contributions

ATA was the principal investigator, participated in the planning and execution of the study, performed data entry and data analysis, and was the main responsible author. AAG, SA, FMA, MAG and DAKG participated in the planning of the study and contributed to the writing process. HSB performed data entry and microbiological work, and contributed to the writing process. HSB participated in the writing. DAKG was the project coordinator and DP participated in planning, data analysis, and writing. All authors read and approved the final manuscript.
